# EU health systems classification: a new proposal from EURO-HEALTHY

**DOI:** 10.1186/s12913-018-3323-3

**Published:** 2018-07-03

**Authors:** Pedro Lopes Ferreira, Aida Isabel Tavares, Carlota Quintal, Paula Santana

**Affiliations:** 1 CEISUC and FEUC, University of Coimbra, Av Dias da Silva, 165, 3004-512, Coimbra, Portugal; 2 CeBER, CEISUC, and FEUC, University of Coimbra, Av Dias da Silva, 165, 3004-512 Coimbra, Portugal; 3 GEGOT and FLUP, University of Coimbra, Largo da Porta Férrea, 3004-530 Coimbra, Portugal

**Keywords:** EU health systems, Factor analysis, Cluster analysis, Health system functions

## Abstract

**Background:**

In accordance the WHO framework of health system functions and by using the indicators collected within the EURO-HEALTHY project, this work aims to contribute to the discussion on the classification of EU health systems.

**Methods:**

Three methods were used in this article: factor analysis, cluster analysis and descriptive analysis; data were mainly collected from the WHO and Eurostat databases.

**Results:**

The most relevant result is the proposed classification of health systems into the following clusters: Austria-Germany, Central and Northern Countries, Southern Countries, Eastern Countries ‘A’ and Eastern Countries ‘B’.

**Conclusions:**

The proposed typology contributes to the discussion about how to classify health systems; the typology of EU health systems allows comparisons of characteristics and health system performance across clusters and policy assessment and policy recommendation within each cluster.

**Electronic supplementary material:**

The online version of this article (10.1186/s12913-018-3323-3) contains supplementary material, which is available to authorized users.

## Background

In 2000, the World Health Organization (WHO) [1] defined a health system as being the set of activities whose primary purpose is to promote, restore and maintain health [1]. This accounts for all formal health services, actions by traditional healers, all use of medication, home care, and actions intended to improve health indirectly by influencing how non-health systems function. Health systems have the responsibility to improve a person’s health, protecting them against the financial cost of illness, as well as treating them with dignity [1, 2].

Existing health systems are a product of, and are influenced by, specific political, historical, cultural and socio-economic traditions. Consequently, they may differ considerably across countries [3]. Indeed, the variety of health systems in Europe has provided ample motivation to those wanting to make comparisons amongst them. Classifying the EU health systems is important for three main reasons: first, it is a rational way to label what is complex; second, classification across health systems allows for international comparisons of not only their characteristics but also their performance; finally, health system classification enables policy assessments and recommendations to be made within each cluster. These are the reasons that have motivated the need for a typology of EU health systems available for the EURO-HEALTHY research project [4].

The EUROHEALTHY project aimed to advance knowledge for those policies with a highest potential to enhance health and health equity across European countries. To achieve this goal, a Population Health Index was computed to evaluate and monitor overall population health, the interactions between health and multiple dimensions at different geographical levels, to foresee and discuss the impact of multilevel policies on population health and geographical health inequalities, and to provide a basis for policy dialogue on health and health equity [4, 5].

From a European policy perspective, considering groups of countries which share similarities in certain aspects falls into alignment with our proposal for a classification of EU health systems.

The classification of health systems further creates the potential to make comparisons and to motivate future health policy assessments and policy recommendations within each health system cluster. The stakeholders of each health system may identify and discuss problems and priorities based on international comparisons of and references to the same cluster. This discussion may be advanced at two levels: within the scope of policy discussions which may be addressed by supra-national institutions, such as the European Commission, or via managerial discussions which allow for potential comparisons of performance across health systems that are similar in their structure as defined here.

A considerable number of tools and analytical instruments have so far been developed and used to classify health systems. However, most proposed typologies include a small number or an incomplete set of EU countries. This may be justified either because the comparable and available data were insufficient or because the typology serves a particular purpose of the author. Few of the suggestions include all or nearly all the EU health systems [6, 7]. A review and historical perspective of the classification of health systems may be found in the work by Bohm et al. [8].

Table 1 presents an overview of some healthcare system typologies. In this table, the work of different authors is listed in chronological order. The typologies proposed are presented according to the criteria and the corresponding countries.

**Table 1:** Overview of some healthcare system typologies

Author (year)	Typology (countries)	Criteria
Field (1973) [20]	Pluralist healthcare (US)	Stewardship; ownership; doctors autonomy
	Health insurance (Western European countries, Japan)	
	National health service system (UK)	
	Socialist healthcare system (USSR, Eastern Europe)	
OECD (1987) [21]	Beveridge model (UK, Nordic countries, Southern European countries, Ireland)	Coverage; funding; ownership
	Bismarck model (Austria, Belgium, France, Germany, Luxembourg, Netherlands)	
	Private insurance (US)	
Donalson and Gerard (1993) [22]	Tax funding (Denmark, Norway, Sweden, UK)	Funding
	Social insurance contributions (France, Germany)	
	Mixed systems (Italy, Spain, Netherlands)	
European Parliament (1998) [3]	Main/supplementary system: Public taxation/private VHI and direct payments (Finland, Greece, Ireland, Italy, Sweden, Spain, UK)	Funding
	Public taxation/direct payments (Denmark, Portugal)	
	Social contributions insurance/private VHI, direct payments, public taxation (Austria, Belgium, France, Germany, Luxembourg)	
	Mixed compulsory social insurance and private voluntary health insurance/public taxation, direct payments (Netherlands)	
WHO (1997) [23]	Beveridge model, mainly taxed based (Denmark, Finland, Iceland, Ireland, Norway, Sweden, UK)	Funding
	Bismarck model, mainly insurance based (Austria, Belgium, France, Germany, Luxembourg, Netherlands, Switzerland)	
	Mixed system: 3 sub-groups are considered: Systems in transition, mainly Bismarkian type (Israel, Turkey)	
	Systems in transformation I from insured to taxed system (Greece, Italy, Portugal, Spain)	
	Systems in transformation II from Semasko to insured system (ex-communist countries)	
Tuohy (1999) [24]	National health service (UK)	Modes of social control: hierarchy; ollegiality; market
	Social insurance (Canada)	
	Private insurance (US)	
Moran (2000) [25]	Entrenched command and state control (Scandinavia, UK)	Consumption; provision; technology
	Supply state (US)	
	Corporatist state (Germany)	
	Insecure command and control state (Greece, Italy, Portugal)	
Freeman (2000) [26], Freeman and Schmidt (2008) [27]	National health service (Italy, Sweden, UK)	Financing; delivery; regulation
	Social insurance system (France, Germany)	
Docteur and Oxley/ OECD (2003) [28]	Public-integrated model (Nordic countries, Italy, Greece, Portugal)	Relations across providers; payers; users
	Public-contract model (Continental European countries, UK)	
	Private insurance/provider (Switzerland, US)	
Thompson et al. (2009) [6]	Social insurance (Austria, Belgium, Czech Republic, Estonia, France, Germany, Lithuania, Luxembourg, Netherlands, Poland, Romania, Slovakia, Slovenia, Bulgaria)	Funding
	Taxed financed (Denmark, Finland, Ireland, Italy, Malta, Portugal, Spain, Sweden, UK)	
	Out-of-pocket payments (Cyprus, Greece, Latvia)	
Wendt (2009) [9]	Health service provision oriented (Austria, Belgium, France, Germany, Luxembourg)	Healthcare expenditure; financing; provision; institutional characteristics
	Universal coverage controlled access (Denmark, UK, Sweden, Italy, Ireland)	
	Low budget restricted access (Portugal, Spain, Finland)	
Wendt, Frisina and Rothgang (2009) [17], Bohm et al. (2013) [29]	National health service (Denmark, Finland, Norway, Sweden, Portugal, Spain, UK)	Financing; provision, regulation
	National health insurance (Ireland, Italy, Canada)	
	Social based mixed (Slovenia)	
	Social Health Insurance (Austria, Germany, Luxembourg, Switzerland)	
	Private healthcare system (US)	
	Statist social health insurance (Belgium, Estonia, France, Czech Republic, Hungary, Netherlands, Poland, Slovakia, Israel, Japan)	
Figueras et al. (1994) [30], Genova (2010) [31]	Northern macro-region (Sweden, Norway, Finland, Denmark, UK, Ireland)	Neighborhood; one common feature
	Center Western macro-region (France, Germany, Austria, Netherlands, Belgium, Luxembourg)	
	Center Eastern macro-region (Poland, Czech Republic, Slovakia, Hungary, Slovenia, Estonia, Lithuania)	
	Southern macro-region (Italy, Spain, Portugal, Greece)	
Joumard et al. (2010) [10]	Private provision and private insurance for basic coverage (Germany, Netherlands, Slovakia, Switzerland)	Institutions; regulations; policies
	Private provision, public insurance for basic coverage, private insurance beyond basic coverage and some gate-keeping (Belgium, France)	
	Private provision, public insurance for basic coverage, little private insurance beyond basic coverage and no gate-keeping (Austria, Czech Republic, Greece, Luxembourg)	
	Public provision and public insurance, no gate-keeping and ample choice of providers (Iceland, Sweden)	
	Public provision and public insurance, gate-keeping, limited choice of providers and soft budget constraint (Denmark, Finland, Portugal, Spain)	
	Public provision and public insurance, gate-keeping, ample choice of providers and strict budget constraint (Hungary, Ireland, Italy, Norway, Poland, UK)	
Reibling (2010) [7]	Financial incentives states (Austria, Belgium, France, Sweden, Switzerland)	Gatekeeping; cost-sharing; provider density; medical technology
	Strong gatekeeping and low supply states (Denmark, Netherlands, Poland, Spain, UK)	
	Weakly regulated and high supply states (Czech Republic, Germany, Greece)	
	Mixed regulation states (Finland, Italy, Portugal)	
EU (2012) [32]	Decentralized (Austria, Italy, Spain)	health funding by Local and Regional Authorities (LRA); power and responsibility by LRA with regard to health-related legislative, planning, and implementation functions; ownership and management of health care facilities by LRA
	Partially decentralized - funding level above EU average (Denmark, Estonia, Finland, Lithuania, Poland, Sweden, Hungary)	
	Partially decentralized - funding level below EU average (Belgium, Czech Republic, Germany)	
	Operatively decentralized - funding level below EU average (Bulgaria, Latvia, Luxembourg, Romania, Slovakia, Slovenia)	
	Operatively decentralized - funding level low or null (Netherlands, UK)	
	Centralized but structured at territorial level (France, Greece, Portugal)	
	Centralized (Cyprus, Ireland, Malta)	

These health system typologies may be based on a single criterion, usually funding, or on several criteria. Moreover, while two criteria necessarily imply a bi-dimensional descriptive analysis, a larger number of criteria require other statistical analyses, such as clustering algorithms, to find homogeneous groups [7, 9, 10]. Although some classifications include the majority of EU countries, none of the more recent classifications includes all 28 EU countries. It has been argued that when going beyond a small-sized comparative case study design, there is a need to reduce complexity by developing a comprehensive framework that describes all cases on the basis of precisely defined dimensions and comparably collected indicators [7]. In the current paper, we propose a classification for the whole set of EU Member States under the WHO’s framework of health systems’ functions and make use of the indicators collected within the EURO-HEALTHY project for the computation of the Population Health Index.

### Conceptual framework

According to the WHO framework [1, 2], every country possesses a health system, fragmented or not, systematically operating or not, focused on three goals: improving the health of the population, responding to citizens’ legitimate expectations, and providing financial protection against the costs of ill-health. Moreover, it is stressed that all three objectives matter in every country, independently of how rich or poor it is or how its health system is organized [1].

In order to achieve these fundamental goals, the WHO proposed four main functions for a healthcare system [1]. These functions are: (i) health service provision, (ii) generation of health resources (investment and training), (iii) health financing, and (iv) stewardship. As noted by the WHO [1], just as the principal objective of a health system is to improve people’s health, the chief function which the system needs to perform is to deliver health services, and the other functions matter in part because they contribute to delivery. Failure to generate resources can put providing services at risk, and services at times are not delivered to potential beneficiaries because the services are underfinanced. Providing services is what health systems *do*; all that health systems can actually do is to deliver specific services or interventions [1]. Resource generation is concerned with the creation of three principal health system inputs: human resources, physical capital, and consumables. The ultimate responsibility for the overall performance of a country’s health system must always lie with government [1].

The four healthcare system functions proposed by the WHO in 2000 [1] have been disaggregated into six functions presented in the report “Everybody’s business: strengthening health systems to improve health outcomes: WHO’s framework for action” [2]. The six functions are: (i) service delivery, (ii) medical products, vaccines and technology, (iii) workforce, (iv) information, (v) financing and (vi) leadership and governance. However, functions (i) - (iv) essentially correspond to a disaggregation of the functions of health service provision and the generation of health resources presented in the 2000 report.

Our classification considers these functions: (i) health service provision, (ii) generation of health resources (investment and training), and (iii) health financing, proxied by some of the indicators collected within EURO-HEALTHY [4, 5]. To build the EU health system classification, we resort to the dimensions of ‘healthcare resources’, ‘healthcare utilization’ and ‘healthcare expenditure’ used to construct the Population Health Index [5]. Other dimensions, associated with areas of concern such as economic and social environment, demographic change, and health outcomes are used to describe the groups of EU health systems.

## Methods

### Data and variables

Data used in this paper were based on the EURO-HEALTHY reference year data available in the Eurostat and WHO HFA/DB databases for the 28 EU countries [11, 12]. Regarding the process of selection of indicators, it started with an extensive review of the literature concerning the whole set of determinants of population health. This was followed by an enchained 2-round Delphi process (51 Consortium experts and 30 stakeholders participated in this exercise) and two meetings and one decision conference of the Project Steering Group [4, 5]. EURO-HEALTHY also produced a protocol providing specifications on how the data needed to be collected, and a web-based platform for data was developed to make data available [4].

The definitions of variables and the acronyms used are the same as those adopted by the EURO-HEALTHY project – Table 2. The three functions of health systems identified by the WHO [1] are proxied by these variables in the following matching way: provision function (HD); resource generation function (MD); and financing function (THE, THEG, and OOP). Regarding provision, the variable used is directly linked with hospital activity; however, because these are chronic conditions, fewer discharges may also mean that these conditions are being prevented and/or managed by primary care services. The second function is proxied by medical doctors per 100,000 inhabitants. Being a supply side variable, it reflects the capacity of health care systems to deliver adequate care to their populations, but, as noted above, although human resources are the most relevant inputs, we should bear in mind what they can provide and how this also depends on physical inputs and consumables. Finally, proxies used for the financing function include not only total health expenditure but also public expenditure and out-of-pocket payments. The breakdown of total health expenditure gives an indication of access to health care as direct payments are a regressive mode of financing and can thus act as relevant barriers to access.

**Table 2:** List of variables used for factor analysis

Acronym	Description	Source
HD	Hospital discharges due to diabetes, hypertension or asthma (per 100,000 inhabitants). It is the formal release of a patient from a hospital after a procedure or treatment. It can refer to inpatients or day cases.	(1)
MD	Medical doctors (per 100,000 inhabitants), health professionals who study, diagnose, treat and prevent illness, disease, injury and other physical and mental impairments in humans through the application of the principles and procedures of modern medicine. They plan, supervise and evaluate the implementation of care and treatment plans by other health care providers and conduct medical education and research activities.	(1)
THE	Total health expenditures per capita (PPS$), i.e. the sum of public and private expenditure on health.	(2)
THEG	Total public health expenditures per capita (PPS$), i.e. the health expenditures from the public sector, including health maintenance, restoration or enhancement paid for in cash or in kind by government entities, transfer payments to households to offset medical care costs and extra-budgetary funds to finance health.	(2)
OOP	Out-of-pocket payments, i.e. private households’ out-of-pocket payment on health (% of total health expenditure), including gratuities and payments in-kind made to health practitioners and suppliers of pharmaceuticals, therapeutic appliances, and other goods and services, whose primary intent is to contribute to the restoration or to the enhancement of the health status of individuals or population groups, and including household payments to public services, non-profit institutions or non-governmental organizations.	(2)

Source: (1) Eurostat database 2012 [11]; (2) WHO/DB-HFA database 2012 [12]

Additional indicators have also been used to describe clusters. These include variables such as GDP per capita and real growth rate, the unemployment rate, mortality rate, life expectancy and DALY’s. Tables 3 and 4 present the definitions for these additional variables.

**Table 3:** List of general socioeconomic variables used

Acronym	Description	Source
GDP	Gross Domestic Product per capita (PPS$), i.e. a measure of the economic activity and of the wealth of a country.	(1)
Real GDP Growth rate	The growth rate of the GDP in terms of volume (%), values determined by the prices of the previous year, and thus computed volume changes are imposed on the level of a reference year so that price movements will not inflate the growth rate.	(2)
Government Debt	General government gross debt (% of GDP, million EUR).	(1)
U	Unemployment rate (%), i.e. the percentage of unemployed people in the active population.	(3)

Source: (1) Eurostat database 2013 [11]; (2) Eurostat database 2012 [11]; (3) Eurostat database 2014 [11]

**Table 4:** List of health outcome indicators used

Acronym	Description	Source
PM	Premature mortality (standardized death rate), i.e. the mortality taking place before the age of 65 (the age limit used in the bulk of international work) in a 3-year period.	(1)
LEB	Life expectancy at birth, i.e. the average number of years that a new-born could expect to live if he/she were to pass through life exposed to the age-specific death rates prevailing at the time of birth, for a specific year, in a given country, territory or geographical area.	(2)
IM	Infant mortality, i.e. the ratio of the number of deaths of children under one year of age during the three-year period to the number of live births in that period.	(2)
DALY	Disability Adjusted Life Years adjusted for differences in the age distribution of the population, and expressed in per 100,000 people. One DALY represents the loss of the equivalent of one year of full health. DALYs for a disease or health condition are the sum of the years of life lost to due to premature mortality (YLL) and the years lived with a disability (YLD) due to prevalent cases of the disease or health condition in a population.	(3)

Source: (1) Eurostat database 2008–2010 [11]; (2) Eurostat database 2013 [12]; Global Health Observatory Data Repository (WHO) 2012 [33]

### Statistical analysis

Our statistical analysis of the data collected followed a 3-step procedure. In the first step, using factor analysis with a varimax with Kaiser normalization for rotation method, we searched for three factors which could express the common variance of the original indicators used as proxies for health systems functions appearing in the WHO framework. In this step, the KMO measure of sampling adequacy was estimated and the Bartlett test of sphericity was performed. The factor analysis was based on the correlation matrix, and the factor scores, to be used in the following cluster analysis, were obtained using the Bartlett method.

The second step was a cluster analysis of the factors found in the previous step for each country in order to identify groups of objects that are similar to each other but different from objects from other groups. The clustering performed here was based on a hierarchical method, which may be visualized in a dendrogram.

The third part of this work was descriptive. It aimed to characterize the clusters found in the previous step. The characteristics used to present the clusters were grouped in socioeconomic characteristics and health outcome indicators.

The statistical software package used for this analysis was the IBM SPSS.20 ®.

## Results

### Step 1: Factor analysis

The correlations among the indicators of provision, resources, and funding are presented in Table 5. As we can observe, most bivariate correlations are not statistically significant, except for those relating to the funding function of health systems.

**Table 5:** Correlations between indicators

	HD	THE	OOP	THEG
MD	0.114 (*p* = 0.614)	0.069 (*p* = 0.726)	0.189 (*p* = 0.335)	0.038 (*p* = 0.846)
HD		−0.411* (*p* = 0.034)	0.320 (*p* = 0.110)	−0.384* (*p* = 0.050)
THE			−0.602** (*p* = 0.001)	0.988** (*p* < 0.001)
OOP				−0.677** (*p* < 0.001)

* / ** statistical significance at 0.05 / 0.01 level

Before proceeding to the factor analysis, the Kaiser-Meyer-Olkin (KMO) measure of sampling adequacy and Bartlett’s test of sphericity were computed. These two tests guaranteed that the sample of data was adequate for factor analysis. The results obtained showed that the KMO measure (KMO = 0.522) was larger than 0.5 (the minimum acceptable value to proceed with factor analysis [13–15], and the Bartlett test rejected the null hypothesis that the correlation matrix was an identity matrix (*p* < 0.001), so indicating that no perfect correlation exists.

Factor analysis assumes an extraction method by the principal component analysis of three factors and a varimax rotation method with Kaiser normalization. These three factors presented in Table 6 correspond to the three health system functions, which were able to explain 92% of the total variance of the initial set of indicators; the third factor contributes with approximately 15% to the explained variance.

**Table 6:** Factor analysis rotated component matrix

	Factor 1	Factor 2	Factor 3
THEG	0.957	0.090	−0.193
THE	0.923	0.141	−0.239
OOP	−0.815	0.295	0.080
MD	0.001	0.976	0.061
HD	−0.227	0.072	0.969
Eigenvalue	2.765	1. 089	0.746
% of Variance	55.3	21.8	14.9

Extraction method: Principal component analysis

Rotation method: Varimax with Kaiser normalization

The first factor, named funding, aggregates the indicators related to health systems financing (THEG, THE, OOP); the second factor includes medical doctors (MD) and it includes resources; and finally, the third factor is represented by hospital discharges (HD) and includes provision.

### Step 2: Cluster analysis

Using the three factors previously found, we clustered the countries by using a hierarchical procedure. This procedure considers the Ward clustering method based on the Euclidean distance. The resulting dendrogram and agglomeration table is presented in Additional file 1: Figure S1 and Additional file 2: Table S1. In the absence of an objective method to optimally select the number of clusters, we determined that 5 clusters represented the reasonable and preferable number to reflect well observed reality, to allow for easy labelling, and to enable international comparisons.

**Fig. 1:**
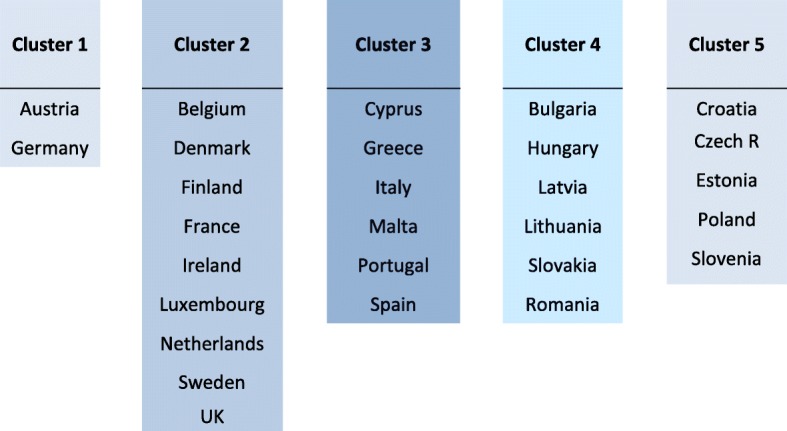
Healthcare systems clusters

The five clusters of countries are displayed in Fig. 1.

Observing how the countries have thus been assembled, clusters of healthcare systems may be labelled as follows: Cluster 1 – Austria-Germany; Cluster 2 - Central and Northern countries; Cluster 3 – Southern countries; Cluster 4 – Eastern countries ‘A’; and Cluster 5 – Eastern countries ‘B’. The clusters labels do not indicate any correlation between geographical positioning and health system characteristics; their only purpose is to simplify identification.

For the three factors, the ‘Austria–Germany’ cluster has positive and higher factor values than the other clusters. The ‘Central and Northern countries’ cluster has a strong funding factor but weaker provision, together with a weaker resources generation. The ‘Southern countries cluster’ aggregates countries with fragile financing and provision but a strong resource generation function. The difference between Eastern countries clusters lies in the provision function. While the ‘Eastern countries ‘A’ cluster has positive factor values, the ‘Eastern countries ‘B’ cluster has negative factor values for provision. Otherwise, with respect to funding and resources, both clusters have high values. The factor scores for each country are displayed in Additional file 3: Table S2.

### Step 3: General description of clusters

Finally, we described the clusters of EU health systems created above according to certain socioeconomic characteristics and health outcome indicators. The figures presented are unweighted averages for each cluster with respect to the selected indicators, with the purpose being to perform a description and a comparison across the clusters.

- Socioeconomic characteristics

Table 7 shows the summary statistics describing the general socioeconomic features of the health systems groups.

**Table 7:** General socioeconomic characteristics

Cluster	GDP ^a^	RGDPgr ^b^	U ^c^	GD ^d^
Austria-Germany	33,521.80	0.30	5.30	80.60
Central and Northern Countries	36,391.18	0.92	8.09	69.20
Southern Countries	22,911.43	1.58	16.63	106.8
Eastern Countries ‘A’	16,749.97	2.43	10.1	44.37
Eastern Countries ‘B’	19,496.12	0.4	9.9	46.56

^a^GDP per capita

^b^RGDPgr – Real GDP growth rate

^c^U - Unemployment rate

^d^GD - Government Debt - General government gross debt, % of GDP, million EUR

Source: Data from Eurostat Database [11]

The wealthiest group of countries is the ‘Central and Northern countries’, which presents the highest GDP per capita whereas the less wealthy clusters are both Eastern countries clusters (‘A’ and ‘B’). For its part, growth in EU economies during this same period of time was seen to be very slow. Nevertheless, the cluster of countries demonstrating a certain level of dynamics was the cluster of ‘Eastern Countries ‘A’ with an average GDP growth over 2% while the ‘Southern Countries’ cluster registered negative economic growth.

The individual labor markets of all EU countries display features which vary significantly. With respect to the unemployment rate – the main indicator of labor market dynamics – the cluster ‘Southern countries’ clearly presents an average unemployment rate higher than the others. The lowest unemployment rates are to be found in the ‘Austria-Germany’ cluster (Table 7).

It is well known that Europe has recently had to face the impact of a significant generalized public debt crisis and, in this context, the ‘Southern Countries’ was identified as having the largest general government gross debt, immediately followed by ‘Austria-Germany’; the lowest values are observed in both Eastern Countries clusters (‘A’ and ‘B’) (Table 7).

- Health outcome indicators

Despite an identical level of development across the 28 EU countries, certain health outcomes are not so similar. Selected health outcome indicators are presented in Table 8. Both Eastern Countries clusters (‘A’ and ‘B’) register lower Life Expectancy at Birth (LEB) than any other cluster.

**Table 8:** Health Outcome Indicators

Cluster	PM^a^	LEB^b^	IM^c^	DALY^d^
Austria-Germany	201.50	81.10	3.38	19,493.50
Central and Northern Countries	199.92	81.34	3.33	19,410.78
Southern Countries	175.88	82.13	3.54	18,113.17
Eastern Countries ‘A’	423.73	75.15	6.22	35,705.33
Eastern Countries ‘B’	316.96	78.24	3.31	23,860.00

^a^PM - Premature mortality - standardized death rate per 100.000 inhabitants

^b^LEB - Life expectancy at birth

^c^IM - Infant mortality

^d^DALY -Disability Adjusted Life Years

Source: Data from Eurostat Database [11]; DALY from Global Health Observatory Data Repository, WHO [33]

There is an observable difference in infant mortality rates across clusters. While the infant mortality rate appears to be similar across four clusters, with the ‘Eastern Countries ‘A’’ cluster presenting a high average value for this indicator.

The life expectancy in both Eastern Countries clusters (‘A’ and ‘B’) is smaller than that in the other clusters. This fact can also be confirmed by the outstanding values of DALY. In contrast, both Eastern countries clusters display the highest values of premature mortality rate whereas ‘Austria-Germany’ and ‘Central and Northern Countries’ clusters have the lowest values.

## Discussion

Taking into consideration both i) the set of health systems functions proposed by the WHO [1] – meant to improve population health, comply with citizens’ expectations, and ensure financial protection – and ii) the need to evaluate and select policies with the greatest potential to improve health and reduce health inequalities in Europe (as reflected in the aims of EURO-HEALTHY), our analysis generated five clusters of countries.

Based on our results, the cluster here identified as ‘Eastern countries ‘A’ shows the worst figures in terms of health. A common shortcoming across these countries points to the low level of financing and thus a lack of financial protection; in addition, the general economic context of these nations is not very favorable, as shown by low GDP per capita, although growth rates seem to indicate some dynamism. Looking at this group of countries as a whole when it comes to European policies might well prove constructive as national/local stakeholders would indeed stand to benefit from sharing their experiences to address common problems.

In terms of health systems’ functions, the cluster ‘Austria-Germany’ stands out with high levels for all factors and quite positive values for socioeconomic and health outcome indicators. The cluster of ‘Central-Northern countries’, on the other hand, shows lower factor values but slightly better health outcomes than ‘Austria-Germany’.

Comparisons across clusters require a certain amount of caution, that is, the results regarding health systems’ functions are influenced by the specific proxies used in this study. Higher hospital discharges can reflect higher levels of hospital activities (in 2015, for instance, hospital discharge rates in general were highest in Austria and Germany [16]). However, hospital activities are affected not only by the capacity of hospitals to treat patients but also by the ability of the primary care sector to prevent avoidable hospital admissions and by the availability of post-acute care settings to provide rehabilitative and long-term care services.

As for human resources, there is some degree of input substitution between medical doctors and other health personnel, namely nurses, meaning that some countries might compensate for a lower number of medical doctors with a higher number of other health personnel. Indeed, in 2015, countries such as Finland, Denmark, Luxembourg and Ireland were among the countries with highest ratios of nurses to doctors [16].

Two Eastern countries clusters were created based on the main difference found with respect to the provision function. But other differences may be found when comparing Eastern countries ‘A’ and ‘B’ clusters. While ‘Eastern countries ‘A’’ has a lower GDP per capita than the ‘Eastern countries ‘B’’, the latter cluster has lower GDP growth than the former.

Finally, the cluster identified as ‘Southern countries’ shows health outcomes close to those seen in ‘Austria-Germany’ and ‘Central-Northern countries’. In terms of health systems’ functions, none emerges with strong factors but several of the countries in the cluster face high out-of-pocket payments and even informal payments leading to less favorable figures in terms of funding. The economic context is characterized by high levels of unemployment and negative growth. Also, despite the good health outcomes, authorities should not lose sight of potential long term effects from the economic crisis that affected Southern countries.

The main objective of this work was, nonetheless, to propose a classification of the EU health systems with no intention to advance conclusions regarding health systems performance. This was done by simply interpreting figures for the indicators collected in the light of the clusters generated. But the framework used in this work and the groups generated may well prove to be quite useful to guide future health policy assessments and policy recommendations within each health system cluster.

Our classification shows some similarities with the clusters proposed by Wendt [17], who also used indicators related with health expenditure (total, public and out-of-pocket payments). In our case, Austria and Germany appear in a separate cluster whereas in his work they come together with the countries that essentially comprise our Central and Northern countries cluster. Two other differences concern the positions occupied by Finland and Italy. In our case, while Finland fits in the Central and Northern countries cluster and Italy fits in the Southern countries cluster, in Wendt’s [17] proposal these countries switch positions. Also, Netherlands and Greece were left out from the clusters in Wendt [9], but in our work, they were placed as expected: in Central and Northern countries and Southern countries, respectively. Comparisons do not apply to Eastern European countries because these were not included in Wendt’s [9] analysis. Comparisons with other studies are even less straightforward, not only due to the number of countries analyzed but also due to the nature of the indicators considered.

Some limitations are reflected in our results, as often occurs with the application of a method. Firstly, the results from factor analysis may be subjective, always depending on the variables included and on the prior choice of the number of factors. For this reason, it does not yield a unique solution [18]. In this work, the decision was taken to create three factors reflecting three health systems’ functions proposed by the WHO [1].

Secondly, cluster analysis algorithms rely on the following initial assumptions with implicit implications [19] on the good performance of the algorithm: (i) spherical clusters, meaning that clusters are not very different in size, density and globular shape, (ii) the prior probability of the k clusters is the same, that is, each cluster has a roughly equal number of observations, and (iii) error variances are cluster invariant and equal across variables. It may be argued as to whether these features are indeed observable in the data because the results are different-sized clusters.

The final limitation to be mentioned concerns the set of indicators selected to create the clusters of EU health systems. The indicators are mainly associated with the supply side, which implies that the classification proposed here is grouping countries according to their health system supply structures. This may have implications both for policy or managerial analytical perspectives as any performance comparison across countries based on our classification must exercise caution as costs and quality are distorting and influencing features in these types of comparisons. Lastly, the set of indicators selected is inevitably subject to variances and/or gaps found in publicly available data.

However, in spite of these potential drawbacks, we defend that an acceptable alternative for an EU-country typology has been achieved. The main contribution of this work is the proposal of a simple, updated and easy classification for 28 EU health systems into four homogeneous groups.

## Conclusions

The purpose of this paper was to contribute to the discussion on the classification of the 28 European health systems. Our proposal was based on three functions of the health systems, namely, provision, resource generation and financing.

The methods used for this analysis have been the factor and the cluster analyses for 28 EU countries and referring to 2012 or the latest available year for the statistical indicator in question. The variables used were hospital discharges, medical doctors, total health expenditure, public health expenditure, and out-of-pocket expenditures, collected within the scope of the EURO-HEALTHY research project.

The 28 EU health systems were clustered into five clusters: ‘Austria-Germany’, ‘Central and Northern Countries’, ‘Southern Countries’, ‘Eastern Countries A’ and ‘Eastern Countries B’. An overview of the clusters reveals that there is a diversity of socioeconomic characteristics across clusters. Health outcomes also vary across clusters but ‘Eastern countries ‘A’ cluster’ tend to register lower levels of life expectancy and higher rates of premature deaths.

This work has provided a review on some of the health system typologies that may be found in the literature. Despite the limitations of the statistical analysis, it is our conviction that the proposal offered in this work contributes positively to the discussion of this topic in the literature and is grounds for potential comparison across the EU health systems. Moreover, the clustering of all EU health systems allows for performance and policy assessments and for policy recommendations within each cluster.

## Additional files

Additional file 1: **Figure S1.** Dendrogram. Dendrogram obtained from SPSS. (DOCX 41 kb)

Additional file 2: **Table S1.** Agglomeration schedule. Table obtained from SPSS. (DOCX 15 kb)

Additional file 3: **Table S2.** Factor scores for each country grouped in clusters. Table displaying factor scores for each country grouped in clusters. (DOCX 14 kb)

## Abbreviations

DALY: Disability Adjusted Life Years
GD: Government debt
HD: Hospital discharges
IM: Infant mortality
LEB: Life expectancy at birth
MD: Medical doctors
OOP: Out-of-pocket expenditures
PM: Premature mortality
RGDPgr: Real GDP growth rate
THE: Total health expenditures
THEG: Public health expenditures
U: Unemployment rate
