# Supplementary figures and images for: EU health systems classification: a new proposal from EURO-HEALTHY
Source: BMC Health Serv Res. 2018 Jul 3;18:511. doi: 10.1186/s12913-018-3323-3 (PMC6029343; doi:10.1186/s12913-018-3323-3)

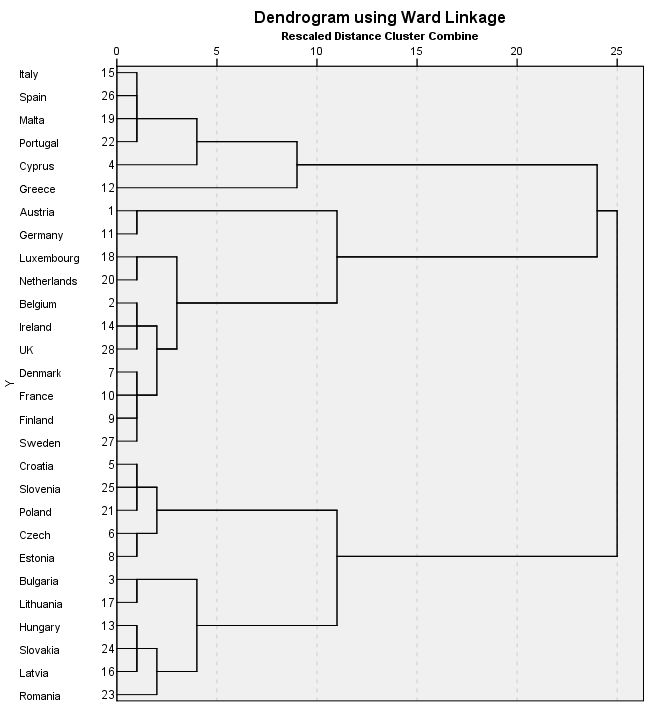

Supplement: Supplementary file 1 — Figure S1. Dendrogram. Dendrogram obtained from SPSS. (DOCX 41 kb) [file 12913_2018_3323_MOESM1_ESM.docx]
