# Supplementary material for: EU health systems classification: a new proposal from EURO-HEALTHY
Source: BMC Health Serv Res. 2018 Jul 3;18:511. doi: 10.1186/s12913-018-3323-3 (PMC6029343; doi:10.1186/s12913-018-3323-3)
Supplement: Supplementary file 2 — Table S1. Agglomeration schedule. Table obtained from SPSS. (DOCX 15 kb) [file 12913_2018_3323_MOESM2_ESM.docx]

| Stage | Cluster Combined | | Coefficients | Stage Cluster First Appears | | Next Stage |
| --- | --- | --- | --- | --- | --- | --- |
|  | Cluster 1 | Cluster 2 |  | Cluster 1 | Cluster 2 |  |
| 1 | 2 | 7 | 2805.103 | 0 | 0 | 15 |
| 2 | 5 | 8 | 7647.243 | 0 | 0 | 9 |
| 3 | 19 | 25 | 14387.022 | 0 | 0 | 6 |
| 4 | 10 | 27 | 21784.689 | 0 | 0 | 15 |
| 5 | 1 | 11 | 30184.313 | 0 | 0 | 22 |
| 6 | 19 | 22 | 49199.273 | 3 | 0 | 14 |
| 7 | 15 | 28 | 74512.713 | 0 | 0 | 19 |
| 8 | 3 | 16 | 102477.077 | 0 | 0 | 13 |
| 9 | 5 | 21 | 133180.384 | 2 | 0 | 20 |
| 10 | 6 | 24 | 166455.661 | 0 | 0 | 12 |
| 11 | 9 | 14 | 208629.531 | 0 | 0 | 19 |
| 12 | 6 | 13 | 254724.303 | 10 | 0 | 20 |
| 13 | 3 | 23 | 310925.918 | 8 | 0 | 17 |
| 14 | 19 | 26 | 371841.124 | 6 | 0 | 16 |
| 15 | 2 | 10 | 437019.768 | 1 | 4 | 22 |
| 16 | 12 | 19 | 515784.423 | 0 | 14 | 18 |
| 17 | 3 | 17 | 648486.005 | 13 | 0 | 23 |
| 18 | 4 | 12 | 846008.099 | 0 | 16 | 24 |
| 19 | 9 | 15 | 1131822.123 | 11 | 7 | 24 |
| 20 | 5 | 6 | 1552635.069 | 9 | 12 | 23 |
| 21 | 18 | 20 | 1982340.364 | 0 | 0 | 25 |
| 22 | 1 | 2 | 2428867.808 | 5 | 15 | 25 |
| 23 | 3 | 5 | 3316094.869 | 17 | 20 | 26 |
| 24 | 4 | 9 | 5379535.211 | 18 | 19 | 26 |
| 25 | 1 | 18 | 8838324.587 | 22 | 21 | 27 |
| 26 | 3 | 4 | 18909671.205 | 23 | 24 | 27 |
| 27 | 1 | 3 | 60410201.116 | 25 | 26 | 0 |
