# Supplementary material for: EU health systems classification: a new proposal from EURO-HEALTHY
Source: BMC Health Serv Res. 2018 Jul 3;18:511. doi: 10.1186/s12913-018-3323-3 (PMC6029343; doi:10.1186/s12913-018-3323-3)
Supplement: Supplementary file 3 — Table S2. Factor scores for each country grouped in clusters. Table displaying factor scores for each country grouped in clusters. (DOCX 14 kb) [file 12913_2018_3323_MOESM3_ESM.docx]

|  | Factor Scores | | |
| --- | --- | --- | --- |
|  | Factor 1 | Factor 2 | Factor 3 |
| Austria | 1.339 | 1.780 | 1.168 |
| Germany | 1.459 | 0.579 | 1.456 |
|  |  |  |  |
| Belgium | 0.684 | -0.465 | -0.429 |
| Denmark | 1.111 | 0.160 | -0.310 |
| Finland | 0.334 | 0.222 | -0.177 |
| France | 1.097 | -0.387 | -0.179 |
| Ireland | 0.341 | -0.525 | -0.588 |
| Luxembourg | 2.175 | -0.516 | -0.199 |
| Netherlands | 1.730 | -0.268 | -0.719 |
| Sweden | 0.810 | 0.631 | -0.178 |
| UK | 0.489 | -1.100 | -0.646 |
|  |  |  |  |
| Cyprus | -1.898 | 0.243 | -2.169 |
| Greece | -0.554 | 3.200 | -0.609 |
| Italy | -0.036 | 0.396 | -1.145 |
| Malta | -0.806 | 0.172 | -0.959 |
| Portugal | -0.651 | 0.913 | -0.693 |
| Spain | -0.402 | 0.367 | -1.109 |
|  |  |  |  |
| Bulgaria | -1.219 | 0.691 | 1.881 |
| Hungary | -0.700 | -0.400 | 0.916 |
| Latvia | -1.218 | -0.255 | 1.100 |
| Lithuania | -0.832 | 0.906 | 1.595 |
| Slovakia | -0.335 | -0.286 | 0.894 |
| Romania | -0.606 | -1.393 | 1.802 |
|  |  |  |  |
| Croatia | -0.467 | -1.016 | -0.191 |
| Czech | -0.245 | -0.113 | 0.165 |
| Estonia | -0.812 | -0.555 | -0.641 |
| Poland | -0.782 | -1.649 | 0.260 |
| Slovenia | -0.006 | -1.330 | -0.295 |
